# Alternative Confidence Interval Methods Used in the Diagnostic Accuracy Studies

**DOI:** 10.1155/2016/7141050

**Published:** 2016-07-11

**Authors:** Semra Erdoğan, Orekıcı Temel Gülhan

**Affiliations:** Department of Biostatistics and Bioinformatics, Faculty of Medicine, Mersin University, 33343 Mersin, Turkey

## Abstract

*Background/Aim*. It is necessary to decide whether the newly improved methods are better than the standard or reference test or not. To decide whether the new diagnostics test is better than the gold standard test/imperfect standard test, the differences of estimated sensitivity/specificity are calculated with the help of information obtained from samples. However, to generalize this value to the population, it should be given with the confidence intervals. The aim of this study is to evaluate the confidence interval methods developed for the differences between the two dependent sensitivity/specificity values on a clinical application.* Materials and Methods*. In this study, confidence interval methods like Asymptotic Intervals, Conditional Intervals, Unconditional Interval, Score Intervals, and Nonparametric Methods Based on Relative Effects Intervals are used. Besides, as clinical application, data used in diagnostics study by Dickel et al. (2010) has been taken as a sample.* Results*. The results belonging to the alternative confidence interval methods for Nickel Sulfate, Potassium Dichromate, and Lanolin Alcohol are given as a table.* Conclusion*. While preferring the confidence interval methods, the researchers have to consider whether the case to be compared is single ratio or dependent binary ratio differences, the correlation coefficient between the rates in two dependent ratios and the sample sizes.

## 1. Introduction

Today in health field, whether an individual is ill or not, the validation of the existence or nonexistence of any kind of illnesses, information about the prognostics of the diagnosed illness, and in some cases the specification of the response to the treatment may be asked. For this reason, many laboratory methods, clinical observations, or many visualization techniques are needed. Many techniques like biochemical tests and bacteria culture tests are evaluated as diagnostics tests, which are highly important in medical field.

Parallel to the advances in science and technology, in medical services, as an alternative to the existing methods, new diagnostic and treatment methods continue to develop. There is no doubt that every work necessitates different tool, way, and method. If the success needs to be achieved, the one suitable for that specific work has to be chosen [1]. Yet there are still many difficulties in the alternative diagnostics tests and treatment applications in medicine and their comparative evaluation. There may be more than one test used in the diagnostics of an illness; yet the absolute ill or absolute healthy diagnostics for the individuals is found by only one of these tests, which is called gold standard method. However, many cases sometimes occur in the testing of some illnesses. The lack of a reference test, the application of reference test's being difficult and expensive, the physical or emotional negative effects of applied methods on patients, and waiting for reference test results to start treatment are only some of these cases stated above. In such cases, as an alternative to the reference test, imperfect reference tests having a certain rate of mistake are preferred. It is a process of decision making to analyze the questions like whether a suggested new diagnostics test is better than the imperfect diagnostics tests in the recognition of patients, or whether one of the visualization techniques is more practical than the other. In this decision making process, the validity of the diagnosis and also the reliability of it in distinguishing the reality are necessary. Besides, it is also crucially important that the diagnosis power of the diagnostics tests is enhanced and the test is applied at the right time. Only in that way would it be possible not to apply unnecessary treatment to the healthy individuals, to apply the necessary treatment to the people carrying the disease, and not to take the healthy individual into an unnecessary operation [2, 3].

While evaluating the applicability and accuracy of the diagnostics tests, some statistical criteria about the validity of the enhanced diagnostics are used. In the most basic definition, the term “diagnostic accuracy” is used to define how close the result of the technique used to differentiate ill and healthy individuals and the true situation are to each other. As for the statistical criteria about the “diagnosis accuracy,” sensitivity, specificity, positive and negative predictive values, odds rates, likelihood ratios, and receiver operating characteristic curve (ROC) can be given [2, 4]. In this study, as the performance criteria of the diagnosis tests, sensitivity and specificity will be taken into consideration. Sensitivity means true positive rate, which is the ability of the diagnosis test to determine the patients correctly among the true patients. Specificity means true negative rate, which is the ability of the diagnosis test to determine the healthy among the true healthy individuals.

A newly improved test needs to be compared with a gold standard test and in such studies, each test is applied on each individual to reduce variability. This kind of studies is called paired design. To decide whether the new diagnostics test is better than the gold standard test/imperfect standard test, the differences of estimated sensitivity/specificity are calculated with the help of information obtained from samples. However, to generalize this value to the population, it should be given with the confidence intervals. Sensitivity and specificity criteria are a proportion of having binary results. For this reason, confidence intervals methods will be used in this study for paired binomial proportions [3, 5]. Furthermore, the aim in this study is to evaluate the Wald confidence interval methods in literature for paired binomial proportions and other confidence interval methods developed as alternatives to them in a clinical application.

## 2. Material and Methods

### 2.1. Statistical Model and General Notations

The methods given for the difference of two dependent sensitivities and the obtained results are similar with the methods given for the two dependent specificities and the obtained results. Thus here, only the difference of two sensitivities and the belonging CI methods will be given. Assume a gold standard test is compared with a newly improved test in the existence of binary results as positive/negative (ill/healthy) in the diagnosis of an illness. When each individual is evaluated with both tests, a two-by-two table representing the results of both tests is below (Table 1). When Table 1 is examined, while cell *a* gives the number of “positives” in both measurements and cell *d* gives the number of “negatives” in both measurements, cells *b* and *c* show the number of discordances as a result of both measurements. Besides, while the proportions are given in the table as *p*_11_ = *a*/*n*, *p*_12_ = *b*/*n*, *p*_21_ = *c*/*n*, and *p*_22_ = *d*/*n*, *p*_1_ and *p*_2_ show the positive proportions and are calculated as (*a* + *c*)/*n* and (*a* + *b*)/*n*, respectively [5–7].

*D*_*i*_ = 1 and *i* = 1,2,… show the true patients for *n* individuals and *X*_*ij*_ = 1  (*j* = 1,2) show the individuals identified as patient as a result of two diagnostics tests. The true sensitivity value for the diagnosis test 1 is shown as se_1_ and the true sensitivity value for the diagnosis test 2 is shown as se_2_. Estimated sensitivity values of the diagnostics tests are calculated as ${\hat{se}}_{1}=\left(a+c\right)/n$ and ${\hat{se}}_{2}=\left(a+b\right)/n$. The null hypothesis of equality of two dependent sensitivities and corresponding alternative hypothesis can be written as [5, 6]

(1) H0:  θ=se2−se1=0,H1:  θ=se2−se1≠0.

The estimating value of the difference between two sensitivities is calculated as $\hat{\theta }={\hat{se}}_{2}-{\hat{se}}_{1}=\left(a+b\right)/n-\left(a+c\right)/n=\left(b-c\right)/n$ and if the CI includes “0,” *H*_0_ hypothesis is accepted; but if the CI does not include “0,” *H*_0_ hypothesis is rejected [5].

The probabilities of discordance of both diagnostics tests are denoted as *p*_12_ and *p*_21_, and estimated overall discordance value is calculated as in [5, 6]

(2) $$\begin{array}{c} \hat{\psi }=p_{12}+p_{21}=\frac{b}{n}+\frac{c}{n}=\frac{b+c}{n}. \end{array}$$

### 2.2. Methods

Methods for the CI estimations can be classified as Asymptotic Intervals (Wald Interval, Continuity Corrected Wald Interval, Wald with Agresti-Min pseudo-frequency adjustment, and Wald with Bonett-Price Laplace Adjustment), Conditional Intervals (Exact conditional method, Mid-*p* Conditional Methods), Unconditional Interval (Unconditional True Profile Likelihood Method), Score Intervals (Wilson Score Interval without Continuity Correction, Wilson Score Interval with Continuity Correction to Score Limits, Wilson Score Interval with Continuity Correction to *ψ*, and Tango Asymptotic Score), and Nonparametric Methods Based on Relative Effect Intervals (Rank-based CI with Normal Approximation, Rank-based CI with *t*-Approximation).

#### 2.2.1. Asymptotic Wald Interval (Wald)

This is a CI method proposed by D. G. Altman in 1991 and is calculated as (3). This method is also based on Central Limit Theorem and normal distribution approach [5, 6]:

(3) $$\begin{array}{c} {CI}_{\alpha /2}\left(\;\hat{\theta }\;\right)=\left[\;\hat{\theta }\pm Z_{\alpha /2}\frac{1}{n}\sqrt{b+c-\frac{{\left(b-c\right)}^{2}}{n}}\;\right]. \end{array}$$

#### 2.2.2. Continuity Corrected Wald Interval (Wald.cc)

With 1/*n* continuity corrected, Asymptotic Wald CI is calculated as in [5]

(4) $$\begin{array}{c} {CI}_{\alpha /2}\left(\;\hat{\theta }\;\right)=\left[\;\hat{\theta }\pm \left(\;Z_{\alpha /2}\frac{1}{n}\sqrt{b+c-\frac{{\left(b-c\right)}^{2}}{n}}+\frac{1}{n}\;\right)\;\right]. \end{array}$$

#### 2.2.3. Wald with Agresti-Min Pseudo-Frequency Adjustment (Wald + *N*, Agresti)

Before using the Wald interval method, Agresti and Min enhanced the efficiency of this method by adding pseudo numbers (*n* = 1,2, 3,4) to the cells observed in Table 1. With this aim, the best performance is observed Wald + 2 according to the results of the simulation applied. According to this, 2 is added overall in every sample and this means adding every cell 1/2  (*n*/4 = 2/4). Thus, $\hat{\theta }=\left(b-c\right)/n=\left(\left(b+1/2\right)-\left(c+1/2\right)\right)/\left(n+2\right)=\left(b-c\right)/\left(n+2\right)$ and new CI method is obtained as in [5, 6, 8]

(5) CIα/2b−cn+2=b−cn+2±Zα/21n+2b+c+1−b−c2n+2.

#### 2.2.4. Wald with Bonett-Price Laplace Adjustment (Bonett-Price)

This is a method developed by Bonett and Price in 2004. According to this method, Laplace estimations are calculated first and then CI is calculated. Laplace estimators are calculated as ${\hat{p}}_{ij}=\left(f_{ij}+1\right)/\left(n+2\right)$. In such a case, ${\hat{p}}_{12}=\left(b+1\right)/\left(n+2\right)$ and ${\hat{p}}_{21}=\left(c+1\right)/\left(n+2\right)$, and Bonett-Price corrected Wald method is calculated as in [8, 9]

(6) CIα/2b−cn+2=b−cn+2±Zα/21n+2b+c+2−b−c2n+2.

#### 2.2.5. Exact Conditional Intervals (Exact.cond)

CI for exact conditional method is defined as in (7). Here, [exact_*l*_(*μ*), exact_*u*_(*μ*)] is Clopper-Pearson Exact CI for *μ* = *π*_2_/(*π*_2_ + *π*_3_) [5, 10–12]:

(7) CI1−α/2θ^=2·exactlμ−1ψ^;2·exactuμ−1ψ^.

#### 2.2.6. Mid-*p* Conditional Intervals (Exact.mid*p*)

This approach is similar to exact conditional confidence interval in terms of calculation steps but here, [mid⁡*p*_*l*_(*μ*), mid⁡*p*_*u*_(*μ*)] has to be used for *μ* and thus CI is formulated as in [5, 12, 13]

(8) CI1−α/2θ^=2·mid⁡plμ−1ψ^;2·mid⁡puμ−1ψ^.

#### 2.2.7. Unconditional True Profile Likelihood Method (Uncond)

In this approach, the maximum likelihood estimator of *ψ* and inverted likelihood ratio test is given as *θ* and is shown as *ψ*_*θ*_. The likelihood function for Θ = 0 is *f*_*ψ*_ = (1 − *ψ*)^*a*+*d*^(*ψ*/2)^*b*+*c*^ and *ψ*_*θ*_ has the biggest value of *ψ*_*θ*_. The likelihood function for Θ ≠ 0 is *f*_*ψ*_ = (1 − *ψ*)^*a*+*d*^((*ψ* + *θ*)/2)^*b*^((*ψ* − *θ*)/2)^*c*^ and $\hat{\psi }$ has the biggest value of $\psi_{\theta }=B+\sqrt{B^{2}-C}$. In this equation, the calculations are as $B=1/2\hat{\psi }+1/2\theta \hat{\theta }$ and $C=\theta \hat{\theta }-\left(a+d\right)/n\theta^{2}$. CI is calculated as in [5]

(9) CI1−α/2θ^=minθ∈−1;1⁡fθfθ≥−z1−α/222;maxθ∈−1;1⁡fθfθ≥−z1−α/222.

The calculation in this equation is defined as $f\left(\theta \right)=\left(a+d\right)\left(\ln\left(1-\psi_{\theta }\right)-\ln\left(1-\hat{\psi }\right)\right)+b\left(\ln\left(\psi_{\theta }+\theta \right)-\ln\left(\hat{\psi }+\hat{\theta }\right)\right)+c\left(\ln\left(\psi_{\theta }-\theta \right)-\ln\left(\hat{\psi }-\hat{\theta }\right)\right)$ [5].

#### 2.2.8. Wilson Score Interval without Continuity Correction (Wilson)

This method is a CI method developed by Newcombe in 1998 and which inverts the score test. The lower limit value of the confidence intervals is given as in (10), and the upper limit value is given as in (11) [5, 8, 12]:

(10) L=θ^−a+bn−l12+u2−a+cn2−2ψa+bn−l1u2−a+cn,

(11) U=θ^+a+cn−l22+u1−a+bn2−2ψa+cn−l2u1−a+bn.

Here, *ψ* is calculated as in (12). Besides, *l*_1_, *l*_2_, *u*_1_, and *u*_2_ are obtained from the quadratic roots of the equations in (13) and (14) [5, 8, 12]:

(12) ψ=0a+b,a+c,b+d,c+d=0ad−bca+ba+cb+dc+da+b,a+c,b+d,c+d≠0,

(13) l1,u1=2a+b+Zα/22±Zα/2Zα/22+4a+b+1−a+b/n2n+Zα/22,

(14) l2,u2=2a+c+Zα/22±Zα/2Zα/22+4a+c+1−a+c/n2n+Zα/22.

#### 2.2.9. Wilson Score Interval without Continuity Correction to Score Limits (Wilson.cc)

This method is a confidence interval similar to “Wilson Score Interval without Correction” but the quadratic roots of both equations are calculated differently. The correction terms in (13) and (14) are obtained by extracting 1/2*n*. Especially in the existence of small samplings, correction term is applied to Wilson score interval method [5].

#### 2.2.10. Wilson Score Interval with Continuity Correction to *ψ* (Wilson.phi)

Especially in the existence of small samplings, a correction term is applied not to the score limits but to *ψ*. This method is a confidence interval similar to “Wilson Score Interval without Correction”, but after *ψ* is calculated not as in (12) but in (15), the lower and upper limit values of CI are calculated by putting in (10) and (11) [5, 12]:

(15) $$\begin{array}{c} \psi =\left\{\begin{array}{cc} 0 & ad\leq bc\\ \max\left(\frac{ad-bc-n}{2},0\right) & ad>bc \end{array}\right\}. \end{array}$$

#### 2.2.11. Tango Asymptotic Score (Tango)

This approach was developed by Tango in 1998 and used as a score test. It has first been used as a dependent samples transformed score test. Later a hypothesis test has been formulized for differences of two rates (as the equality of two rates or the superiority of one of the rates). The confidence interval of the difference between two sensitivity values is given as in [5, 8, 14, 15]

(16) $$\begin{array}{c} T\left(\;\frac{n}{\theta }\;\right)=\frac{b-c-n\theta }{\sqrt{n\left[2{\hat{p}}_{21}+\theta \left(1-\theta \right)\right]}}. \end{array}$$

Here ${\hat{p}}_{21}$ is the maximum likelihood estimator of *p*_21_ and is formulized as ${\hat{p}}_{21}=\left(\sqrt{B^{2}-4AC}-B\right)/2A$. It is calculated as *A* = 2*n*, *B* = −*b* − *c* + (2*n* − *b* + *c*)*θ*, and *C* = −*cθ*(1 − *θ*). Lower limit and upper limit values of Tango asymptotic score confidence interval for Θ are calculated as *T*(*n*/*L*) = *Z*_*α*/2_ and *T*(*n*/*U*) = −*Z*_*α*/2_ [8, 14, 15].

#### 2.2.12. Rank-Based CI with Normal Approximation (np.nv)

Nonparametric confidence intervals are based on ranked data and here asymptotic normal approximation is used. The detailed explanations of the method used have been put forward by Lange and Brunner in 2012 and calculated as in [5, 16]

(17) $$\begin{array}{c} {CI}_{1-\alpha /2}\left(\;\hat{\theta }\;\right)=\left[\;\hat{\theta }\pm Z_{1-\alpha /2}\frac{{\hat{\sigma }}_{\theta }}{\sqrt{N}}\;\right]. \end{array}$$

#### 2.2.13. Rank-Based CI with *t* Approximation (np.*t*)

It has been put forward by Brunner and Munzel in 2000 for small sample sizes and is calculated as in [5, 17]

(18) $$\begin{array}{c} {CI}_{1-\alpha /2}\left(\;\hat{\theta }\;\right)=\left[\;\hat{\theta }\pm t_{\hat{f},1-\alpha /2}\frac{{\hat{\sigma }}_{\theta }}{\sqrt{N}}\;\right]. \end{array}$$

The degrees of freedom for the quartile of *t*-distribution are calculated as $\hat{f}={\left({\hat{\sigma }}_{1}^{2}+{\hat{\sigma }}_{2}^{2}\right)}^{2}/\left(\left({\hat{\sigma }}_{1}^{2}+{\hat{\sigma }}_{2}\right)/\left(N-1\right)\right)$. ${\hat{\sigma }}_{1}^{2}$ and ${\hat{\sigma }}_{2}^{2}$ here show the diagonal elements of covariance matrix [5, 17].

## 3. Results

### 3.1. Clinical Application

In this study, the data used by Dickel et al. in diagnostics study has been taken as samples. According to this study, a total of 790 patients have applied the strip patch test (SPT) as an alternative to patch test (PT) accepted as gold standard test in allergies diagnostics and they have been recorded as being nonallergic. Three different main components as Nickel Sulfate (Ni), Potassium Dichromate (Cr), and Lanolin Alcohol (La) have been used to examine the accuracy of the tests and confidence intervals for differences of the two dependent sensitivities have been calculated for these tests [18]. 122 patients have been observed as being sensitive to Ni, 28 patients to Cr, and 8 patients to La and cross tables of situations concordance and discordance according to both diagnostics tests for each allergen are given in Table 2. It has been calculated that the difference of sensitivity of 122 patients sensitive to Ni is 0.164 and 95% CI is 0.087–0.241, the difference of sensitivity of 28 patients sensitive to Cr is 0.250 and 95% CI is 0.090–0.410, and the difference of sensitivity of 8 patients sensitive to La is 0.125 and 95% CI is −0.104–0.354.

The result of all CI methods is given in Table 3. When the table is analyzed, for 3 different parameters, the same results have been taken in point estimations of differences with all methods except for Agresti's intervals.

For all three parameters, the methods having the narrowest CI have been observed in conditional confidence interval methods and exact.mid*p* method. Conditional confidence interval methods are not affected by the sample size like other methods. When examined in terms of Ni, while CI methods supply similar results, Wald.cc is seen to have a relatively wider CI compared to the other methods. When examined from the point of Cr, there are differences among the confidence intervals of the methods and the methods having the wider confidence interval are Wald.cc and Np.*t* confidence interval methods. However, when examined from the point of La, as the CI includes “0” in all methods, *H*_0_ hypothesis is accepted and the differences between the two sensitivities are not meaningful. No matter which CI method is used, it can be said that SPT is a very good test for Ni and Cr but that it is not such a distinguishing test for testing La.

## 4. Discussion

It has been noticed by some researchers that Wald method, traditional asymptotic confidence interval for the differences between the values of two sensitivities/specificities, is not enough in terms of some evaluation criteria and many CI methods have been developed as an alternative to this method [5, 6, 8, 9, 12, 14, 19, 20].

Newcombe has designed a simulation study about the asymptotic, conditional, unconditional, and score confidence intervals and the performances of these confidence interval methods have been evaluated. According to this, Newcombe advises the researchers to use conditional confidence interval methods (exact cond. and exact mid*p*.) in the cases of one rate and unconditional confidence interval (uncond) and Wilson score confidence interval methods in the cases of dependent rate differences [12].

Wenzel and Zapf have compared different CI methods for the differences between two dependent sensitivities/specificities. When simulation results are examined according to sample size, for all the cases, it has been observed that Tango and nonparametric confidence interval methods (np.nv, np.*t*) give the best results. Besides, the performances of confidence interval methods dependent on the correlation coefficient (between 0.20 and 0.80) between the two sensitivity values have been studied in these simulations. According to this, it has been observed that the performances of Wald cc. exact cond. and Wilson.cc methods are conservative in all scenarios and the methods go worse as the correlation value increases and that exact mid*p*, Agresti, and Wilson.phi methods are conservative independent of the correlation. While uncond method is slightly anticonservative in all scenarios, it goes worse as the correlation increases; it has been stated that Wilson, Tango, and np.*t* methods start as slightly anticonservative but they show a slightly conservative case as the correlation value increases. Wenzel and Zapf, based on all of the possible scenarios they were planning, recommend the researchers to use Wald, Tango, and np.*t* confidence interval methods among confidence interval methods of difference between two dependent sensitivity studies [5].

Agresti and Min have developed Wald confidence interval method with difference modifications and called that Wald + *N* method. In their study, they have tried Wald, Wald + 2, and score confidence intervals for different combinations and have expressed that Wald + 2 methods have a narrower confidence interval method compared to score confidence interval for small samples [6].

Fagerland et al. have made a simulation study on the performances of asymptotic, unconditional, and score confidence interval methods. They have showed that when asymptotic confidence intervals are examined, the performances of Wald and Wald.cc methods are very weak in small samples (*n* = 15), and as sample number increases (*n* = 40), though not very successful, they have a slightly better performance. Besides, while Agresti CI performance value falls below the nominal level 95% in small samples, performance value in Bonett-Price CI falls below the nominal level as the sample number increases. They have also emphasized that Wilson and Tango score interval methods are quite conservative in small samples, that Wilson confidence interval has a narrower confidence interval compared to Tango confidence interval, that Bonett-Price confidence interval has a narrower confidence interval compared to Wilson and Tango score confidence interval, and that unconditional confidence interval method has a wider confidence interval compared to the other methods [8].

Bonett and Price have studied the performances of asymptotic and score confidence intervals as an alternative in small samples for dependent proportion differences. In their studies, they express that classical Wald method show a weak performance, that Tango method is better than Wald.cc method, and that Agresti method has a better performance than Tango and Wald.cc methods [9].

Tango has compared conditional (uncond), unconditional (exact cond, exact mid*p*), and Tango methods and has put forward that Tango method has a more conservative confidence interval for small samples and that in other conditions it shows a better performance. He has also put forward that, besides Tango method, uncond method has shown a better performance in large samples, too [14].

Tang et al. has compared conditional and score confidence interval methods for single rate and small samples. In their studies, they have put forward the fact that conditional confidence interval methods have shown better performance than score confidence interval methods. Another research of Tang et al. (2010) has found that the performances of Tango and Wilson score methods are better in small sample sizes with the comparison of confidence intervals for the differences between two dependent proportions, advising the researchers to use these methods. Tang et al. have found that the performances of Tango and Wilson score methods are better in small sample sizes and have advised the researchers to use these methods [19, 20].

When studied in terms of point estimations, our clinical data show similar results to all other confidence interval methods except for Agresti method and it has been observed that the only method that is not affected by the sample size is conditional confidence interval methods.

It is true that, from the CI methods, Wald methods are preferred by the researchers because of having easier calculation steps compared to the other methods. While preferring the confidence interval methods, the researchers have to consider whether the case to be compared is single ratio or dependent binary ratio differences, the correlation coefficient between the rates in two dependent ratios and the sample sizes.

According to the simulation results, in the literature, when a single ratio comparison has been made, no matter what the sample size is, in the comparisons belonging to the dependent ratio comparison of the conditional confidence interval methods (exact cond., exact mid*p*.), unconditional confidence interval method and Wilson and Wilson phi confidence interval method of the score confidence interval methods should be preferred [5, 6, 8, 9, 12, 14, 19, 20]. Besides, in the cases where dependent ratio differences exist, without looking at the sample sizes, Tango score confidence interval and nonparametric confidence interval methods (np.nv, np.*t*) are seen to give the best results. The use of Wald and np.nv methods depending on the correlation coefficient between the two sensitivity values is advised [5].

## Figures and Tables

**Table 1:** A two-by-two table representing concordance/discordance of both diagnostic tests.

	Diagnostic test 1 (gold standard)		Total
	Positive	Negative	
Diagnostic test 2 (new test)			
Positive	*a* (*p*_11_ = *a*/*n*)	*b* (*p*_12_ = *b*/*n*)	*a* + *b* (*p*_2_ = (*a* + *b*)/*n*)
Negative	*c* (*p*_21_ = *c*/*n*)	*d* (*p*_22_ = *d*/*n*)	*c* + *d*

Total	*a* + *c* [*p*_1_ = (*a* + *c*)/*n*]	*b* + *d*	*n*

**Table 2:** A table representing concordance/discordance of both diagnostic tests of SPT and PT only for patients sensitive to Ni, Cr, and La.

	PT		Total
	+	−	
Nickel Sulfate (Ni)			
SPT			
+	59	23	82
−	3	37	40

Total	62	60	122

Potassium Dichromate (Cr)			
SPT			
+	10	7	17
−	0	11	11

Total	10	18	28

Lanolin Alcohol (La)			
SPT			
+	0	1	1
−	0	7	7

Total	0	8	8

**Table 3:** The results of all confidence interval methods for different sample sizes.

	Nickel Sulfate (Ni) (*n* = 122)		Potassium Dichromate (Cr) (*n* = 28)		Lanolin Alcohol (La) (*n* = 8)	
	se_1_ = 0.508	se_2_ = 0.672	se_1_ = 0.357	se_2_ = 0.607	se_1_ = 0.000	se_2_ = 0.125
	*θ*	CI	*θ*	CI	*θ*	CI
Asymptotic intervals						
Wald	0.164	(0.087, 0.241)	0.25	(0.090, 0.410)	0.125	(−0.104, 0.354)
Wald.cc	0.164	(0.079, 0.249)	0.25	(0.054, 0.446)	0.125	(−0.229, 0.479)
Agresti	**0.161**	(0.084, 0.238)	**0.23**	(0.068, 0.398)	**0.100**	(−0.170, 0.370)
Bonett-Price	0.164	(0.083, 0.240)	0.25	(0.056, 0.411)	0.125	(−0.234, 0.434)

Conditional intervals						
Exact.cond	0.164	**(0.085, 0.203)**	0.25	**(0.045, 0.250)**	0.125	**(−0.119, 0.125)**
Exact.mid*p*	0.164	**(0.093, 0.200)**	0.25	**(0.076, 0.250)**	0.125	**(−0.113, 0.125)**

Unconditional interval						
Uncond	0.164	(0.090, 0.245)	0.25	(0.111, 0.428)	0.125	(−0.142, 0.445)

Score intervals						
Wilson	0.164	(0.086, 0.238)	0.25	(0.086, 0.388)	0.125	(−0.215, 0.471)
Wilson.cc	0.164	(0.083, 0.241)	0.25	(0.070, 0.400)	0.125	(−0.294, 0.533)
Wilson.phi	0.164	(0.085, 0.239)	0.25	(0.071, 0.400)	0.125	(−0.215, 0.471)
Tango	0.164	(0.090, 0.247)	0.25	(0.099, 0.434)	0.125	(−0.240, 0.471)

Nonparametric methods based on relative effects						
np.nv	0.164	(0.087, 0.241)	0.25	(0.087, 0.413)	0.125	(−0.120, 0.370)
np.*t*	0.164	(0.087, 0.241)	0.25	(0.083, 0.47)	0.125	(−0.171, 0.421)
